# CCAAT/Enhancer-Binding Protein Homologous (CHOP) Protein Promotes Carcinogenesis in the DEN-Induced Hepatocellular Carcinoma Model

**DOI:** 10.1371/journal.pone.0081065

**Published:** 2013-12-05

**Authors:** Viviana Scaiewicz, Avital Nahmias, Raymond T. Chung, Tobias Mueller, Boaz Tirosh, Oren Shibolet

**Affiliations:** 1 Institute for Drug Research, School of Pharmacy, Faculty of Medicine, Hebrew University of Jerusalem, Jerusalem, Israel; 2 Liver Unit, Division of Medicine, Hadassah-Hebrew University Medical Center, Jerusalem, Israel; 3 Gastroenterology Division, Massachusetts General Hospital and Harvard Medical School, Boston, United States of America; 4 Department of Gastroenterology and Rheumatology, Section of Hepatology, University Hospital Leipzig, Leipzig, Germany; 5 Liver Unit, Department of Gastroenterology, Tel Aviv Medical Center and Tel-Aviv University, Tel-Aviv, Israel; National Institutes of Health, United States of America

## Abstract

**Background and Aims:**

C/EBP homologous protein (CHOP) plays pro-apoptotic roles in the integrated stress response. Recently, a tumor suppressive role for CHOP was demonstrated in lung cancer via regulation of tumor metabolism. To explore the role of CHOP in hepatocarcinogenesis, we induced hepatocellular carcinoma (HCC) in wild type (wt) and CHOP knockout (KO) mice using the carcinogen N-diethylnitrosamine (DEN).

**Results:**

Analysis of tumor development showed reduced tumor load, with markedly smaller tumor nodules in the CHOP KO animals, suggesting oncogenic roles of CHOP in carcinogen-induced HCC. In wt tumors, CHOP was exclusively expressed in tumor tissue, with minimal expression in normal parenchyma. Analysis of human adenocarcinomas of various origins demonstrated scattered expression of CHOP in the tumors, pointing to relevance in human pathology. Characterization of pathways that may contribute to preferential expression of CHOP in the tumor identified ATF6 as a potential candidate. ATF6, a key member of the endoplasmic reticulum stress signaling machinery, exhibited a similar pattern of expression as CHOP and strong activation in wt but not CHOP KO tumors. Because HCC is induced by chronic inflammation, we assessed whether CHOP deficiency affects tumor-immune system crosstalk. We found that the number of macrophages and levels of IFNγ and CCL4 mRNA were markedly reduced in tumors from CHOP KO relative to wt mice, suggesting a role for CHOP in modulating tumor microenvironment and macrophage recruitment to the tumor.

**Conclusion:**

Our data highlights a role for CHOP as a positive regulator of carcinogen-induced HCC progression through a complex mechanism that involves the immune system and modulation of stress signaling pathways.

## Introduction

Hepatocellular carcinoma (HCC) is the fifth most common cancer and the third cause of cancer-related deaths worldwide [1]. Chronic hepatic inflammation and cirrhosis is responsible for 90% of HCC cases. Current standard treatments for HCC include liver transplantation and surgical resection, local ablative therapies including radiofrequency ablation and transarterial chemoembolization, and targeted therapy with the tyrosine kinase inhibitor, sorafenib. These strategies are often ineffective and are accompanied by a high incidence of recurrence and poor prognosis for the majority of patients with HCC [2]. The underlying molecular mechanisms of inflammation-induced carcinogenesis are under intensive research but have been only partially elucidated.

The endoplasmic reticulum (ER) is the cellular organelle responsible, among other functions, for preparing and directing proteins into secretory pathways via its folding properties. Under homeostatic conditions, the ER folding capacity matches the load of its client proteins. However, under certain conditions this homeostasis is disturbed resulting in the accumulation of unfolded or misfolded proteins, referred to as ER stress. An ER-to-nucleus signaling pathway, collectively called the unfolded protein response (UPR), emanates from the ER to relieve cells of the stress condition. The mammalian UPR is regulated by three ER trans-membrane sensors: the inositol requiring enzyme 1 (IRE1), the double-stranded RNA-activated protein kinase–like ER kinase (PERK), and the activating transcription factor 6 (ATF6) [3]. Each sensor controls a downstream signaling pathway that contributes to reducing ER stress and restoring homeostasis. Activation of the UPR, when transient, leads to attenuation of translation, mRNA and ER protein degradation, increased autophagy and increment of the ER folding capacity by synthesis of new chaperone proteins. However, when the stress persists, programmed cell death ensues [4].

Various mechanisms connect the UPR to the apoptotic machinery. A key regulator of ER stress-induced apoptosis is C/EBP homologous protein (CHOP). This 29 kDa protein has been identified in the DNA damage inducible transcript genetic screen, as belonging to the growth arrest DNA damage (GADD) protein family. Although this transcription factor cannot bind DNA directly because of the presence of proline and glycine residues in its basic region that disrupt its DNA binding activity, it can regulate gene expression by forming hetero-dimers with other proteins from the C/EBP family or other transcription factors, acting as an activator or inhibitor of gene transcription [5].

Under normal conditions, CHOP is minimally expressed and can be found in the cytoplasm. In response to DNA damage, ER stress or other stress responses, CHOP is induced and translocates to the nucleus. CHOP is an effector of the PERK and ATF6 arms of the UPR, and has been shown to regulate apoptosis by mechanisms that include induction of oxidative stress, disturbing iron homeostasis, down-regulation of the anti-apoptotic protein Bcl2 and up-regulation of the death receptor 5 (DR5) [6]–[8]. A recent study utilizing chromatin immunoprecipitation sequencing (ChIP-seq) techniques, demonstrated that CHOP promotes ER stress-mediated apoptosis primarily by enhancing protein synthesis and oxidative stress. Direct binding of CHOP to gene promoters that participate in apoptosis was not observed [9]. Furthermore, nuclear localization of CHOP was shown to directly regulate activation of genes associated to cell movement, growth and proliferation, suggesting a more diverse role of CHOP in cellular processes [10], [11].

The role of ER stress in cancer is controversial. UPR signaling in endothelial cells was demonstrated to promote angiogenesis by controlling VEGF expression [12], [13]. Studies in primary cells demonstrated that the PERK pathway is critical for transformation, suggesting that UPR promotes tumorigenesis [14]. The role of ER stress-mediated apoptosis in cancer has not been widely explored, either. One recent observation, using a RAS-driven lung cancer mouse model, indicates that CHOP serves as a barrier for tumor development, primarily by promoting cell death when nutrients become a limiting factor for tumor growth [15].

In the present study we explored the role of CHOP in diethylnitrosamine (DEN)-induced HCC by utilizing wt and CHOP KO mice. Unexpectedly, deletion of CHOP resulted in significantly smaller tumors, suggesting that CHOP operates as an oncogene in HCC, rather than a tumor suppressor gene as reported for lung cancer. The IRE1 and PERK arms of the UPR were minimally activated. However, the ATF6 arm showed marked tumor-specific activation. We further show that CHOP is activated in human tumors of various origins. Macrophage infiltration into CHOP KO tumors and inflammatory cytokine and chemoattractant levels were reduced relative to the wt controls. Our data reveal a new role for CHOP in hepatic tumorigenesis and highlights the duality of ER stress in cancer.

## Results

### CHOP Promotes Tumor Progression

**Figure 1:**
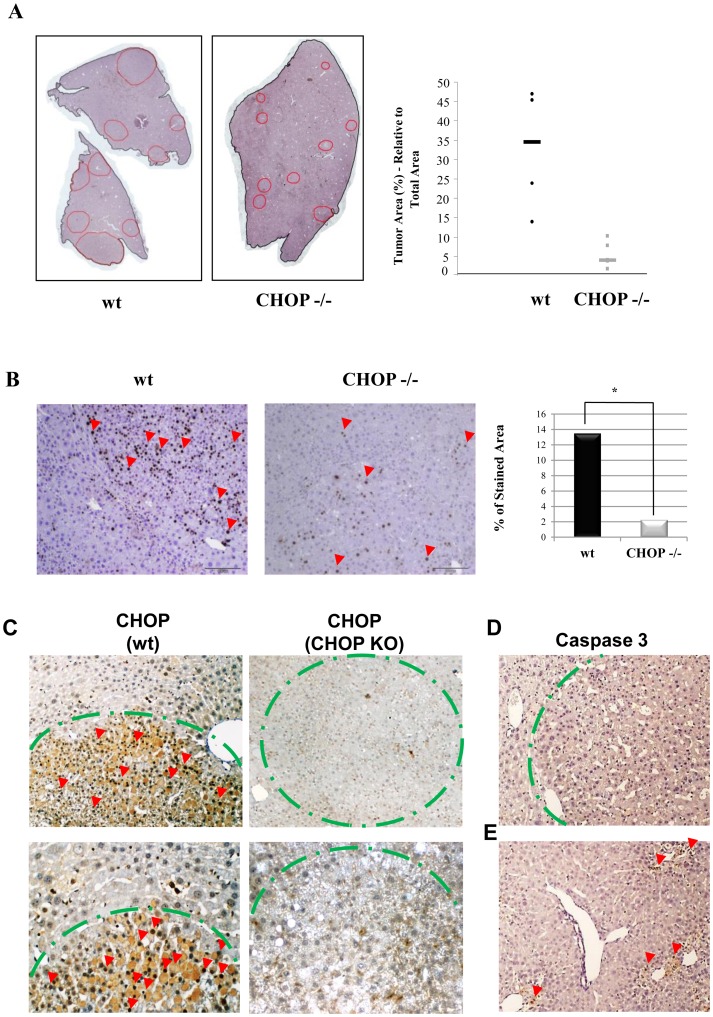
CHOP KO mice develop smaller HCC nodules. A. Shown is a representative development of HCC in WT and CHOP KO livers (red lines mark tumors nodules). Tumor area was calculated as a percentage of total analyzed liver area (n = 8) (right panel). B. Cell cycle activation was assessed by Ki67 staining (red arrows indicate positive staining). Slides were scanned and stained area was quantified automatically using ARIOL software (n = 8). C. Histological sections of WT and CHOP KO HCC were stained for CHOP (red arrows), the green dotted line demarks tumor borders. D. Histological sections of DEN induced HCC were stained for activated caspase 3 (upper panel), E. staining for active caspase 3 24 h following DEN injection was used as a positive control (lower panel).

To explore the role of CHOP in HCC initiation and progression, 12 wt and 12 CHOP KO mice were injected intraperitoneally with DEN at the age of 15 days. We used an accelerated protocol of DEN-induced tumors as previously described by Huang et al, and mice were sacrificed at the age of 5 months [16]. Livers were removed and tumors were assessed macroscopically and histologically. For both genotypes, tumors were found in 11 of the 12 DEN-treated mice. The number of tumor nodules present in a defined area of liver tissue was compared between wt and CHOP KO mice. No significant differences were found in the number of tumor nodules. However, nodule size/total area was approximately 10 fold smaller in the CHOP KO compared to wt mice, as quantified using ImageJ software (3.93%±3.3% and 34.57%±15.8% respectively) (Figure 1A).

Because tumor size is related to cell proliferation capacity, we stained the tissue for the nuclear protein Ki67, a marker of cell cycle activation. Our results show that expression of Ki67 was significantly higher in tumors from wt as compared to CHOP KO mice (13.5% vs. 2.27% of total cells, as quantified by ARIOL, p<0.05 (Figure 1B). These results indicate that CHOP promotes tumor cell proliferation. To verify the expression of CHOP in the DEN-induced wt tumors, sections that contain the borders between the tumor and the normal parenchyma were stained by immunohistochemistry (IHC) for CHOP. Remarkably, CHOP expression was clearly confined to the nuclei of the tumor cells, and was absent in the adjacent parenchymal cells. Interestingly, despite robust expression of CHOP in the tumor (Figure 1C), we did not observe evidence of apoptosis, as indicated by the absence of active caspase 3 expression (Figure 1D). As a positive control for apoptosis we analysed the livers 24 h after DEN-induced DNA damage (Figure 1E). We conclude that CHOP is specifically expressed in HCC tumors in a manner that does not promote apoptosis. Expression of CHOP plays a pro-tumorigenic role.

### Nuclear CHOP is Observed in Human Adenocarcinomas

**Figure 2:**
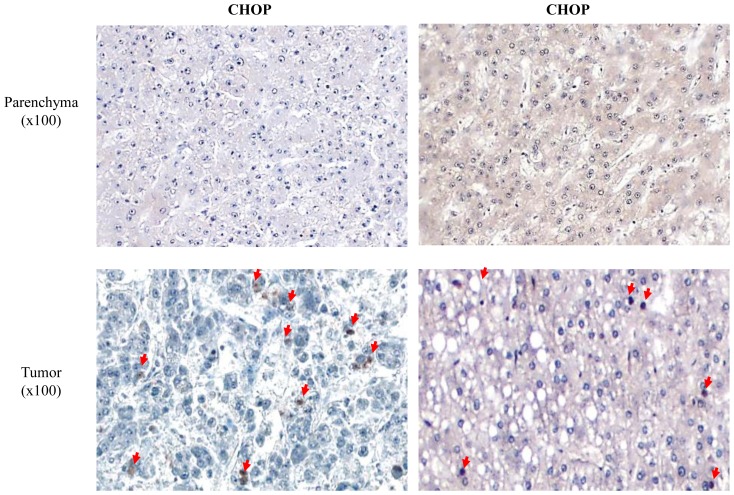
Nuclear CHOP in observed in human HCC. Human samples were stained for CHOP, nuclei were visualized by hematoxylin staining (red arrows).

To investigate the relevance of our mouse data to human pathology, CHOP expression was assessed in human HCC. We obtained HCC biopsies from 9 patients. Nuclear staining for CHOP was observed in 6 of the 9 samples. In contrast, CHOP was not expressed in adjacent human parenchyma, supporting its exclusive tumor expression (Figure 2).

Further analysis of human tumors demonstrated that CHOP expression was not restricted to HCC. We found robust CHOP expression in prostate and pancreas adenocarcinomas. Again, CHOP was predominantly expressed in the tumor tissue and minimally in the surrounding normal parenchyma (Figure S1 in File S1). These data suggest that CHOP may also play a pro-tumorigenic role in various other types of human carcinomas.

### UPR is Mildly Activated in DEN-induced HCC

**Figure 3:**
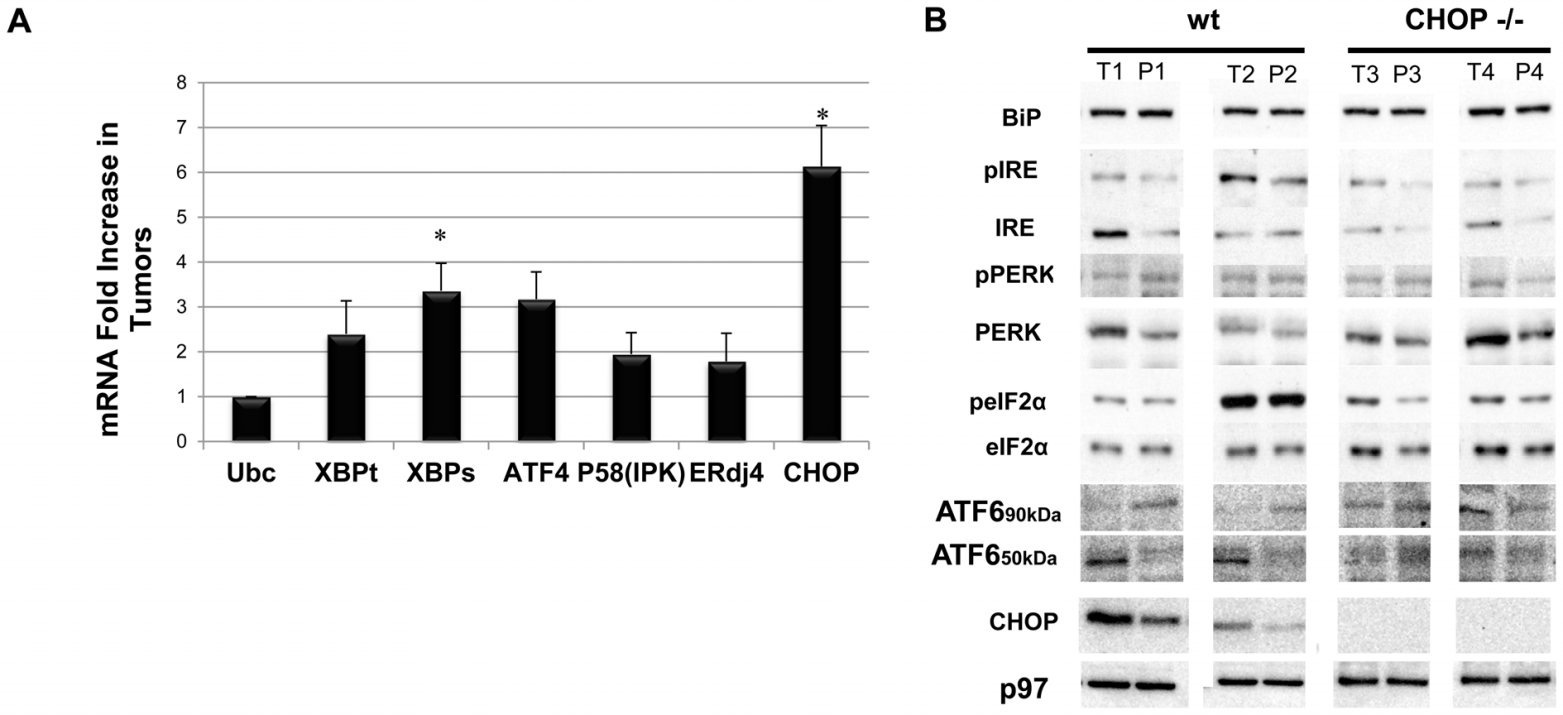
Differential activation of UPR pathways in the DEN-induced HCC mouse model. A. Real-time PCR analyses of UPR target genes. Tumor gene expression was compared to its corresponding parenchyma (n = 8, p<0.01). B. Western Blot analysis of total liver extract for different UPR proteins (n = 5, p<0.05). 20 µg were loaded for Bip, eIF2, CHOP, p97 analysis; 40 µg were loaded for all other UPR markers.

Several mechanisms contribute to CHOP expression. CHOP is transcriptionally induced downstream of ATF4, the hub protein of the integrated stress response (IRS) [17]. In addition, CHOP is a direct target of ATF6 [18], and eIF2α phosphorylation on Serine 51 was also shown to promote CHOP translation [19]. We assessed the contribution of the different arms of the UPR to CHOP expression in the DEN-induced tumors in comparison to their parenchyma in a pair-wise tumor/parenchyma manner. CHOP mRNA levels were increased 6-fold in the tumors as compared to parenchyma, implicating transcription control rather than translation or degradation as the main contributor to CHOP expression in the tumors (Figure 3A). This was corroborated by a lack of increase of PERK phosphorylation or its eIF2α substrate in the tumors. Interestingly, in the CHOP KO animals, eIF2α phosphorylation was induced in the tumors, supporting a role of CHOP in the feedback of protein synthesis homeostasis (Figure 3B).

Next, we assessed the existence of ER stress conditions in the tumors. The ratios of phosphorylated IRE1 to total IRE1 levels were similar between wt tumors and parenchyma (Figure 3B). Furthermore, there was a non-significant increase in XBP-1 mRNA splicing and lack of induction of the XBP-1 specific target genes, ERdj4 and p58IPK [20]. These findings point to a minimal contribution of the IRE1 arm of the UPR to the induction of CHOP expression in the wt tumors. We were not able to detect the endogenous ATF4 protein levels using various commercial antibodies. Analysis of ATF4 mRNA indicated a moderate increase. However, since ATF4 expression is regulated primarily at the translation level [21], our data cannot confirm nor exclude the contribution of ISR to CHOP expression.

ATF6 is converted into a transcription factor by regulated intra-membrane proteolysis in response to ER stress, liberating its 50 kDa cytoplasmic N-terminus fragment for transcription induction [22]. Immunoblotting analysis showed robust cleavage of ATF6 in the wt tumors, as compared to reduced expression in the parenchyma (Figure 3B). We therefore decided to explore the activation of ATF6 by IHC in the DEN-induced tumors, as well as in the various human adenocarcinomas. An isolated strong nuclear staining was observed in tumor tissue (Figure S1 & S2 in File S1). Our data identifies ATF6 as a putative inducer of CHOP in tumors. The mechanisms governing ATF6 selective activation in the tumors in contrast to the mild activation of the other two arms of the UPR remain to be elucidated.

### CHOP Promotes Inflammation and Macrophage Infiltration into the DEN-induced Tumors

**Figure 4:**
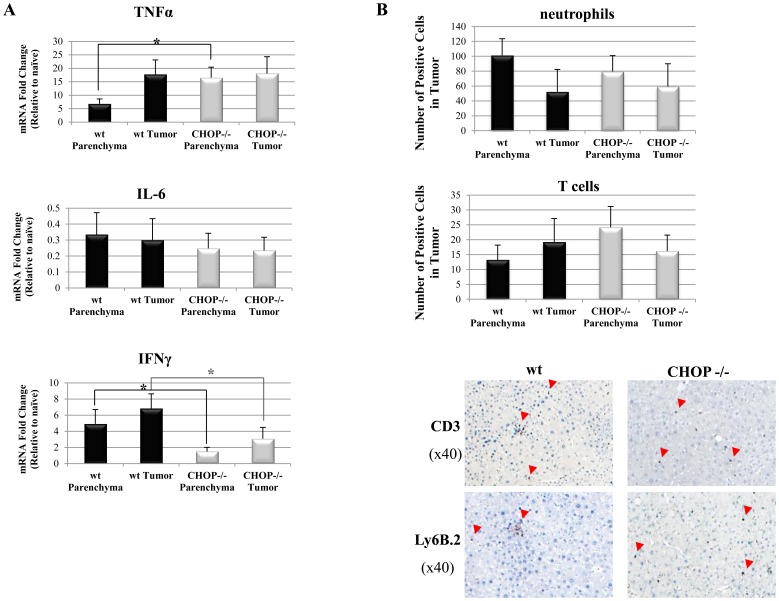
Cytokine levels and immune cell infiltration into DEN-induced HCC. A. Analysis of cytokines mRNA levels in tumor and parenchyma of WT and CHOP−/− mice (n = 5, p<0.01 for statistical significance). B. Histological sections of liver tissue slides were stained for T cells (anti-CD3) and neutrophils (anti-Ly6B.2). The total number of CD3 or Ly6B.2 positive cells in wt and CHOP −/− tumor and parenchyma were counted (n = 5 for each genotype).

**Figure 5:**
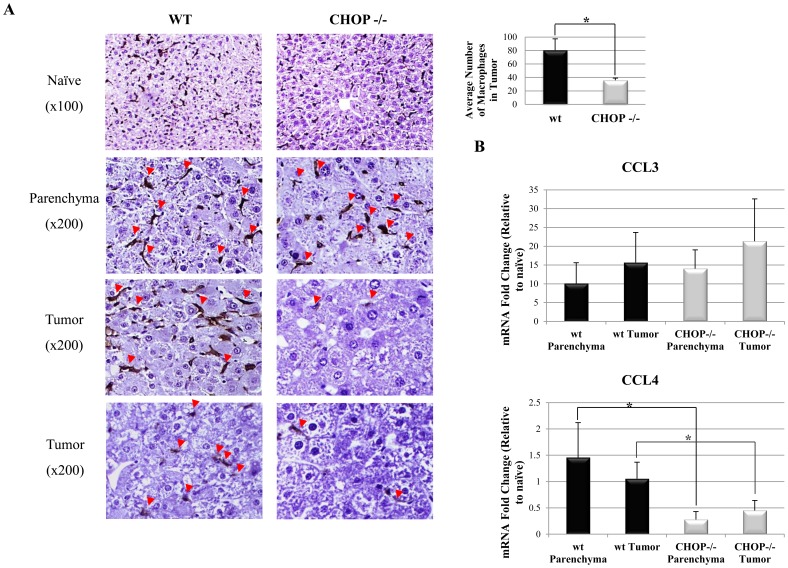
Chemokine levels and macrophages infiltration into DEN-induced HCC. A. Histological sections of WT and CHOP KO livers were stained for the macrophage marker F4/80. Total number of F4/80 positive cells (red arrows) were counted in seven different tumor zones in each sample (n = 5 of both genotypes, p<0.05). B. Analysis of mRNA expression of chemokines in tumor and parenchyma of WT and CHOP−/− mice.

Clinically, HCC usually develops on the background of chronic hepatic inflammation, induced by hepatitis viruses, alcohol, fat or exposure to other hepatotoxic substances. DEN-induced tumors share similar pathogenesis, in which pro-inflammatory cytokines such as IL-6 and TNFα promote tumor development [23]. A common observation in HCC and other solid tumors is the infiltration of macrophages [24]–[30]. We therefore assessed the level of pro-inflammatory cytokines and infiltration of immune cells in tumors of wt and CHOP KO mice. Interferon gamma (IFNγ) mRNA level was significantly lower (Figure 4A) while those of IL-6 and TNFα were similar in wt and CHOP KO tumors respectively. Analysis of immune cell infiltrates demonstrated similar numbers of CD3-positive T lymphocytes and Ly6B.2-positive neutrophils in wt and CHOP KO livers (Figure 4B). Wt tumors showed a rich macrophage presence as detected by F4/80 staining. In contrast, a marked decrease in the number of infiltrating macrophages was observed in CHOP KO tumors (80 macrophages/zone vs. 36 macrophages/tumor zone respectively p = 0.012) (Figure 5A). These data are in agreement with the reduced IFNγ levels, as macrophages infiltration into the DEN tumors is dependent on intact IFNγ signalling [31]. Finally, we analysed the mRNA levels of CCL3 and CCL4, chemokines involved in macrophage recruitment, by qPCR. CCL4 levels were reduced in the CHOP KO as compared to wt controls (Figure 5B). These data demonstrate a combined defect in the inflammatory response in the CHOP KO tumors manifested by reduced numbers of macrophages, key promoters of inflammation-induced-carcinogenesis.

## Discussion

HCC is a leading cause of morbidity and mortality. Although much is known about the role of inflammation in hepatic carcinogenesis, the specific role of ER stress, and individual UPR regulator proteins in this process are largely unexplored. CHOP is a key pro-apoptotic member of the UPR. Recently its role in modulating other cellular processes has come to light, including involvement in hepatocyte proliferation and inflammatory cell movement [32].

Under normal conditions, CHOP is expressed at a low level in the cytoplasm where it has been suggested to exist in a non-functional state. However, following homeostatic perturbations such as ER and oxidative stress induction, CHOP levels markedly increase and the protein becomes active following nuclear translocation, a process that requires accessory molecules [10]. Following nuclear translocation, CHOP undergoes hetero and homo-dimerization and acquires DNA binding capacities in trans, which allow it to promote or inhibit gene expression. The context in which CHOP is expressed largely determines cellular commitment and fate. Recent data shows that under strong ER stress conditions inflicted by tunicamycin treatment in fibroblasts, CHOP primarily partners with ATF4. This leads to the dysregulation of protein synthesis and promotion of oxidative stress, cumulatively promoting apoptosis [9]. In the liver, under a challenge of tunicamycin or proteasome inhibition, CHOP suppresses lipid synthesis by outcompeting C/EBPα and C/EBPβ DNA binding [33]. However, if instead of interfering with protein folding and degradation, mice are fed with methionine-choline deficient diet, which promotes the development of liver steatosis and nonalcoholic fatty liver disease (NAFLD), CHOP protected against NAFLD in a mechanism that involved exacerbated apoptosis of liver macrophages [34].

In the present study we assessed the role of CHOP in a well-characterized carcinogen-induced HCC model by using CHOP KO and wt mice. While similar numbers of malignant nodules were observed in CHOP KO, the nodules were markedly smaller. Furthermore, CHOP was exclusively expressed in the tumor cells, but not in the normal parenchyma (Figure 1). This exclusive expression pattern was also observed in human tumors of various origins, but was not universal and not as pronounced as in the murine model (Figure 2 and Figure S1 in File S1). This different histological appearance might be related to the fact that the human samples were all taken during liver resection and represent advanced disease, most usually on the background of cirrhotic changes. While in the mice histological analysis was performed early in the disease course when cirrhotic changes have not yet developed. Furthermore, tumor induction by DEN is a singular event and is not fully reminiscent of human tumors in which multiple pathways are activated over prolonged periods, some of which may inhibit or reduce CHOP expression. All these suggest that multiple mechanisms may impinge on CHOP expression in the human tissues and thus affect its heterogeneous expression.

CHOP activation was not universal within the human samples, suggesting that other oncogenic pathways induce hepatic carcinogenesis in a CHOP-independent manner. The tumors we examined were from patients with HCC in cirrhotic livers of various underlying causes (HCV, HBV, NASH etc.). Due to the limited number of samples, this study cannot assess the contribution of specific underlying liver disease etiologies to CHOP activation in human tumors.

The observation that CHOP, a pro-apoptotic protein supports HCC tumorigenesis was unexpected. Cell death plays a dual role in the DEN-induced carcinogenesis process. During the early stages of development, apoptosis initiates tissue reconstruction after the DNA damage inflicted by the carcinogen, a process important for tumor initiation. In this phase apoptosis contributes to local inflammatory conditions and release of tumor promoting cytokines, such as TGFα. Later, when micro-tumors are formed, apoptosis limits tumor progression, especially under hypoxic conditions or lack of nutrients. Accordingly, over-expression of the anti-apoptotic gene Bcl2 inhibits DEN-induced liver carcinogenesis, primarily due to attenuation of tumor initiation [35]. Thus, it may be that the absence of CHOP interferes with the immediate apoptosis required for HCC induction in the DEN model [36]. The fact that a similar number of tumor nodules develop in wt and CHOP KO mice, suggest that there may be a tumor initiating event that is CHOP-independent but once formed, lack of CHOP is not permissive for growth. It may be that CHOP is more an accelerant of HCC rather than an inducer and is only involved in the tumor progression phase.

In the analysis of factors that may induce CHOP preferentially in the tumor, we characterized ER stress as a likely factor. In the DEN model, tumors develop over a 5–9 month period, the conditions of ER stress, if triggered are chronic in nature, rather than acute as explored experimentally in the aforementioned studies. Accordingly, we have not observed robust activation of the PERK and IRE1α pathways of the UPR, both of which strongly affect liver metabolism (Figure 3) [37], [38]. In contrast, the nuclear fragment of ATF6 was expressed preferentially in the wt tumors and much less in the parenchyma. Furthermore, ATF6 cleavage was milder in CHOP KO, alluding to a feedback mechanism that may exist between CHOP and ATF6 in tumors (Figure 3). Thus our data implicates ATF6 as a factor upstream to CHOP. It is conceivable that the suppression of HCC progression in the CHOP KO mice may also be related to complex feedback loops that CHOP relays to the UPR machinery.

In the DEN model, HCC development is affected by the immune system through secretion of cytokines (primarily TNFα and IL-6) and lipid eicosanoid mediators [39], all of which are robustly produced by Kupffer cells, the liver-resident macrophages. It is therefore not surprising that Kupffer cells are strong instigators of HCC [24]. We therefore analyzed the presence of immune cells in wt and CHOP KO tumors. While neutrophils and T cells were present in similar numbers in both tumor genotypes (Figure 4), macrophages were virtually absent from the CHOP KO tumors (Figure 5). It is not clear what upstream signals contribute to this phenotype. Quantitative PCR analysis pointed that the macrophage attractant chemokine CCL4 was diminished in the CHOP KO tumors as a possible upstream signal controlled by CHOP (Figure 5). Cytokine profiling indicated changes in IFNγ expression as well (Figure 4A). Whether these are the sole regulators of the differential macrophage infiltration into wt and CHOP KO tumors is not known. The data presented here counter-intuitively highlights CHOP as a potential oncogenic factor in HCC. We show that CHOP regulates hepatic carcinogenesis via mechanisms involving UPR signaling, primarily a crosstalk with ATF6, and an effect on macrophage recruitment into the tumors. Our data marks CHOP as a possible target for drug development in HCC.

## Materials and Methods

### Ethics Statement

Animal care and experiments were approved and conducted in accordance with the guidelines of the Authority for Biological and Biomedical Models of the Hebrew University, approval number MD-10-12531-4. The Institutional Animal Care and Use Committee (IACUC) specifically approved the animal part of the study. Collection of human tissues was approved by the Ethics committee of the Tel-Aviv Medical Center, Tel-Aviv Israel. Because tissues were unidentified, according to the declaration of Helsinki, no written informed consent was necessary. The ethics committee specifically waived the need for written informed consent from the participants. Approval number is 0352-12-TLV.

### Animal Model

C67BL/6J and CHOP−/− (on a C57BL/6J background) animals were purchased from Jackson laboratory (Bar Harbor, ME, USA), and bred in a pathogen free facility. Fourteen days after birth, animals were treated with a single intra-peritoneal injection of diethylnitrosamine (99% purity, Sigma-Aldrich, cat#73861, MO, USA) at a dose of 10 µg per gram diluted in 0.9% saline. Starting two weeks after DEN injection, mice were treated with biweekly injections of 1,4-Bis-[2-(3,5-dichloropyridyloxy)]benzene (TCPOBOP) (98% purity, Sigma-Aldrich, cat#T1443, MO. USA) at a concentration of 3 µg per gram diluted in corn oil as previously described [40]. Five months after birth, animals were sacrificed, RNA and protein extracted, and tissue was preserved in 4% paraformaldehyde for paraffin fixation.

### RNA Isolation and Quantitative PCR

Total RNA from tissue was isolated using TriReagent (Sigma-Aldrich) according to the manufacturer’s protocol. cDNA was generated using High Capacity cDNA Reverse Transcription kit (Applied Biosystem, CA, USA) from 2 µg of RNA with random hexameric primers. Quantitative PCR was carried out using Kapa syber fast qPCR kit (KapaBiosystems, MA,USA) using the primers specified in Table S1 in File S1. Ubiquitin C was used as the house keeping reference gene for all qPCR analyses. All reactions were performed at annealing temperature of 58°C.

### Western Blotting

Liver tissue was lysed in RIPA lysis buffer. Protein samples were then separated by SDS–polyacrylamide gel electrophoresis and transferred to PVDF membranes. Subsequently, membranes were blocked and probed with specific antibodies (Table S2 in File S1) followed by secondary horseradish peroxidase-conjugated antibodies. Reactive bands were visualized using an enhanced chemiluminescence reaction and subjected to densitometric analysis relative to p97.

### Immunohistochemistry

Tissue was fixed in 4% formaldehyde for 16 h and dehydrated in 70% ethanol. Tissue was then embedded in paraffin and 10 µm sections were used for analysis. Sections were deparaffinized in histo-clear (Kaltek, Padova, Italy), and then rehydrated in serial washes with ethanol gradient. Retrieval was performed in 10 mM citrate buffer, pH = 6 at 120°C and 1.8 atm for 3 minutes. Secondary antibody kit MACH3 (Biocare Medical, CA, USA) was used for detection and amplification of first antibody signal. After substrate reaction, nuclei were stained using haematoxylin. Samples were then dehydrated in serial washes with ethanol gradient and sealed.

### Staining Quantification and Tumor Measuring

Tumors and total area was calculated using ImageJ software (rsbweb.nih.gov/ij) applied to scanned images from haematoxylin and eosin staining (augment × 5). IHC positive staining was measured by automated image analysis quantification (Ariol-SL50).

### Statistical Analysis

Statistical significance between groups was assessed by one-way ANOVA adjusted for multiple comparisons and tested for normal distribution. Data are expressed as means ± standard deviations. Triplicate determinations were performed in all the real-time experiments, and all experiments were repeated at least three times. A p-value equal or less than 0.05 was considered to be statistically significant.

## Supporting Information

File S1 **Table S1. List of antibodies. Table S2. Primers list for qPCR. Figure S1. ATF6 is activated in human HCC. Figure S2. CHOP and ATF6 are activated in different human adenocarcinomas.**
(PDF)
