# Supplementary material for: CCAAT/Enhancer-Binding Protein Homologous (CHOP) Protein Promotes Carcinogenesis in the DEN-Induced Hepatocellular Carcinoma Model
Source: PLoS One. 2013 Dec 5;8(12):e81065. doi: 10.1371/journal.pone.0081065 (PMC3855209; doi:10.1371/journal.pone.0081065)
Supplement: File S1 — Table S1. List of antibodies. Table S2. Primers list for qPCR. Figure S1. ATF6 is activated in human HCC. Figure S2. CHOP and ATF6 are activated in different human adenocarcinomas. (PDF) [file pone.0081065.s001.pdf]

Table S1. List of antibodies

Antibody Name	Method	Company & Catalogue number
ATF6	WB & IHC	Abcam (#11909)
BiP	WB	Abcam (#21685)
CHOP	WB	Cell Signaling (#L63F7)
eIF2α	WB	Cell Signaling (#9721)
p eIF2α	WB	Cell Signaling (#9722)
F4/80	IHC	AbD Serotec (#mca497r)
GADD153	IHC	Santa-Cruz Biotechnology (#sc-575)
Ki67	IHC	Thermo Scientific (94-RM-9106)
IRE	WB	Cell Signaling (#3294)
pIRE	WB	Abcam, Cambridge, UK, (#48187)
PERK	WB	Cell Signaling (#3192)
pPERK	WB	Cell Signaling (#3179)

Table S2. Primers list for qPCR

Gene	Primer Forward	Primer Reverse
atf4	ccttcgaccagtcgggtt	ctgtcccggaaaaggcat
ccl3	actgcctgctgcttctcctaca	aggaaaatgacacctggctgg
ccl4	ttcctgctgtttctcttacacc	ctgtctgcctcttttggt
ccl5	agatctctgcagctgccctca	ggagcacttgctgctggtgtag
chop	ctggaagcctggtatgag	cagggtcaagagtagtga

erdj4	ataaaagccctgatgctg	gccattggtaaaagcact
ifnγ	atgaacgctacacactgcatc	ccatccttttgccagttcctc
il-6	tctataccacttcacaagtcgga	gaattgccattgcacaactcttt
p58ipk	tttactgccgcaagacta	ctggggttagatttgagc
tnfα	ggcaggtctactttggagtcattg	acattcgaggctccagtgaattcgg
xbp1 (spliced form)	gagtccgcagcaggtg	gtgtcagagtccatggga
xbp1 (total)	aagaacacgcttgggaatgg	actccccttggcctccac
ubc	cagccgtatatcttcccagact	ctcagagggatgccagtaatcta

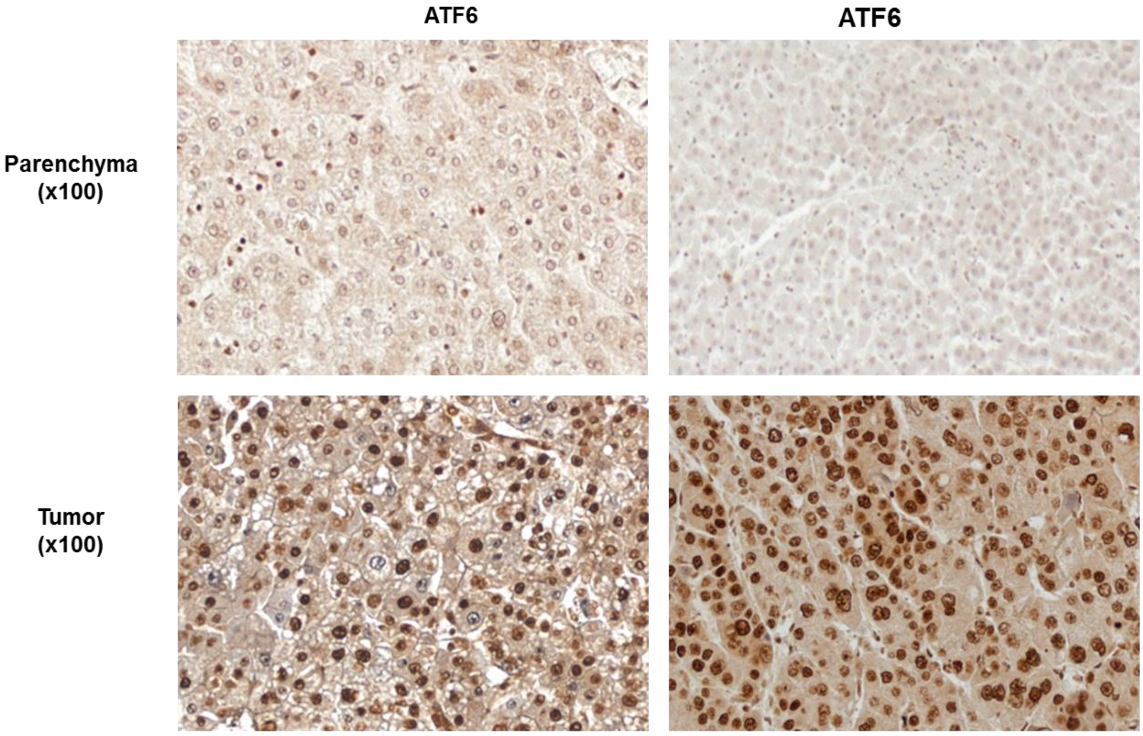

Figure S1. ATF6 is activated in human HCC. Representative samples from liver tumor resected from patients, stained for ATF6.

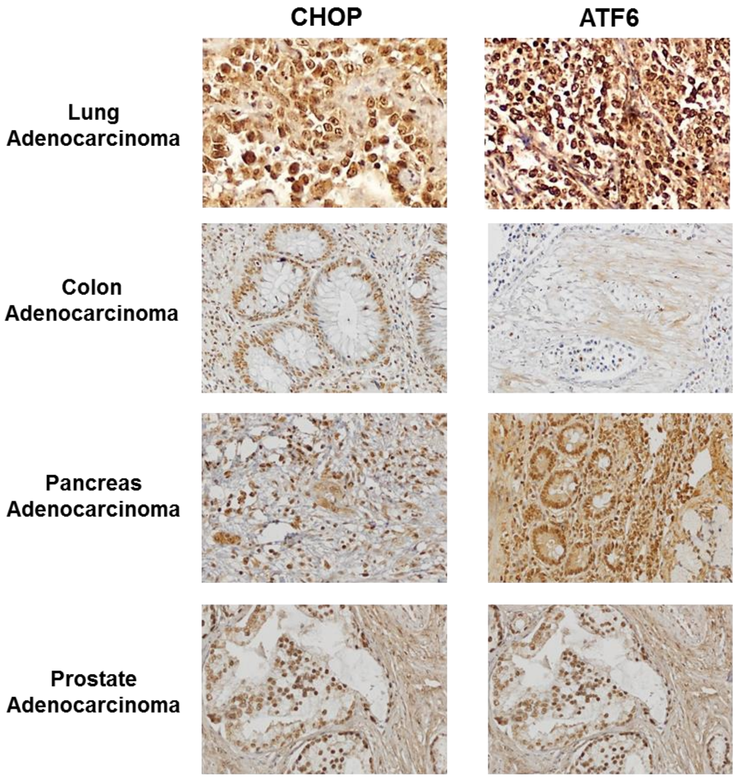

Figure S2. CHOP and ATF6 are activated in different human adenocarcinomas. Representative samples from lung, colon, pancreas and prostate adenocarcinomas show activation of CHOP (left) and ATF6 (right).
